# Supplementary material for: Transcriptional activity mediated by β-CATENIN and TCF/LEF family members is completely dispensable for survival and propagation of multiple human colorectal cancer cell lines
Source: Sci Rep. 2023 Jan 6;13:287. doi: 10.1038/s41598-022-27261-0 (PMC9822887; doi:10.1038/s41598-022-27261-0)
Supplement: Supplementary file 1 — Supplementary Information. [file 41598_2022_27261_MOESM1_ESM.pdf]

Supplementary Information for:

Transcriptional activity mediated by β-CATENIN and TCF/LEF family members is completely dispensable for survival and propagation of multiple human colorectal cancer cell lines

Janna Fröhlich, Katja Rose, and Andreas Hecht

This file contains:

Supplementary Figures S1 - S7

Supplementary Tables S1 - S4, S16 - S18

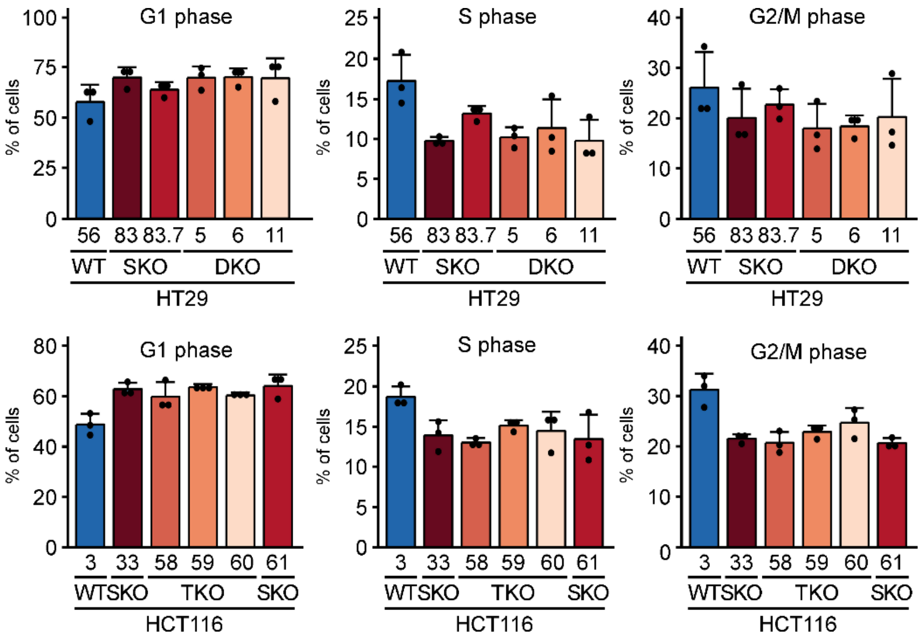

Suppl. Fig. S1 Complete absence of TCF/LEF expression does not aggravate the proliferation defect resulting from TCF7L2-deficiency. To examine differences in cell cycle distribution, HT29 and HCT116 cells with the genotypes indicated were stained with propidium iodide and analyzed by flow cytometry. The proportions of cells in different cell cycle phases are depicted by the bar plots. Each dot represents an individual measurement; the error bars indicate SD (n=3). Statistical significance of differences in the percentages of cells in different cell cycle phases was not analyzed for this series of experiments due to different group sizes of WT, SKO, DKO and TKO cell clones (see Methods).

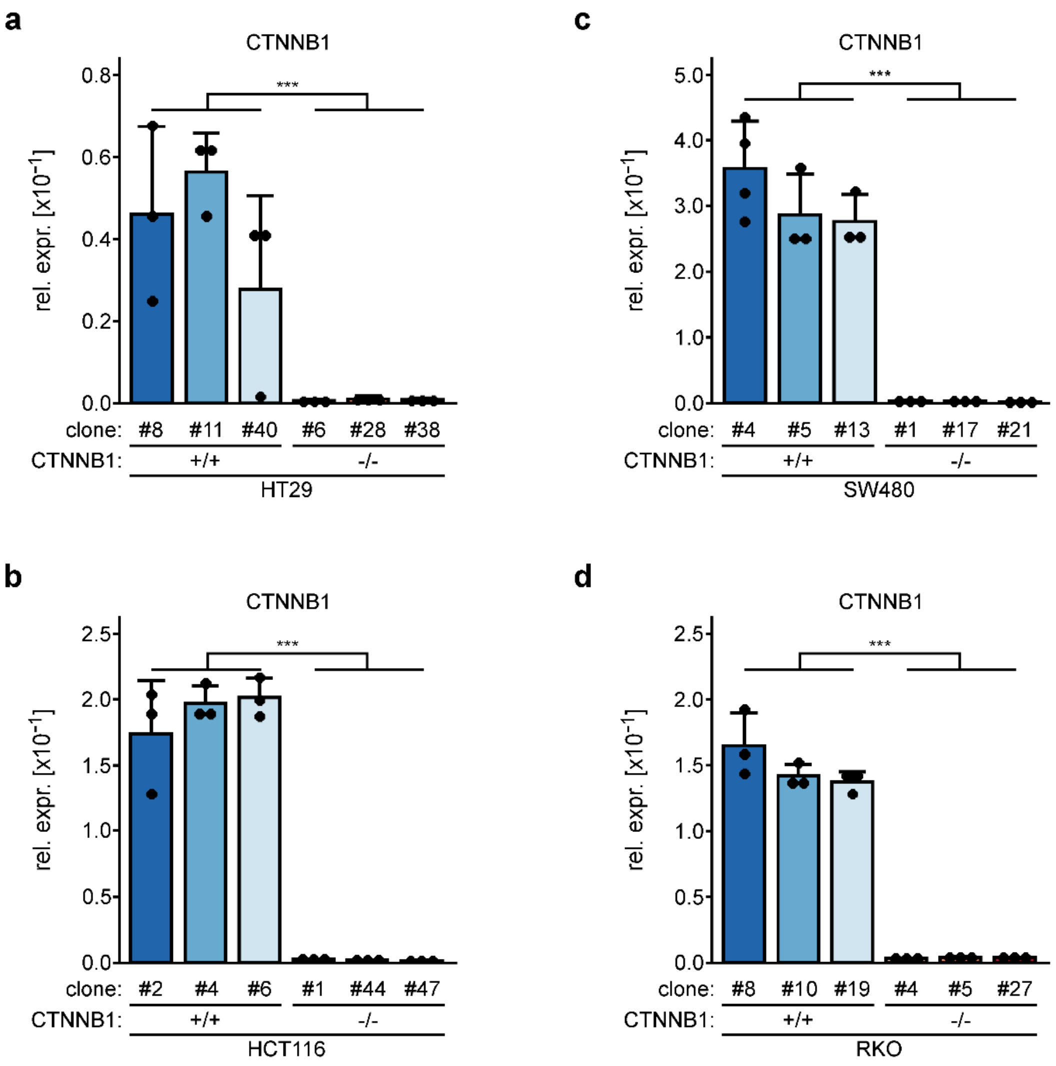

Suppl. Fig. S2: Deletion of *CTNNB1* exon 7 results in vastly reduced RNA expression. a-d Transcript levels of the *CTNNB1* gene were analyzed by qRT-PCR in HT29 (**a**), HCT116 (**b**), SW480 (**c**), and RKO (**d**) cell clones with biallelic wildtype and mutant *CTNNB1* genes. *CTNNB1* transcript levels were normalized to those of *GAPDH*, and are displayed as relative expression (rel. expr.). The bar graphs summarize the expression data; error bars indicate SD (n≥3). Each dot represents an individual measurement. For statistical analyses, LMM was performed.

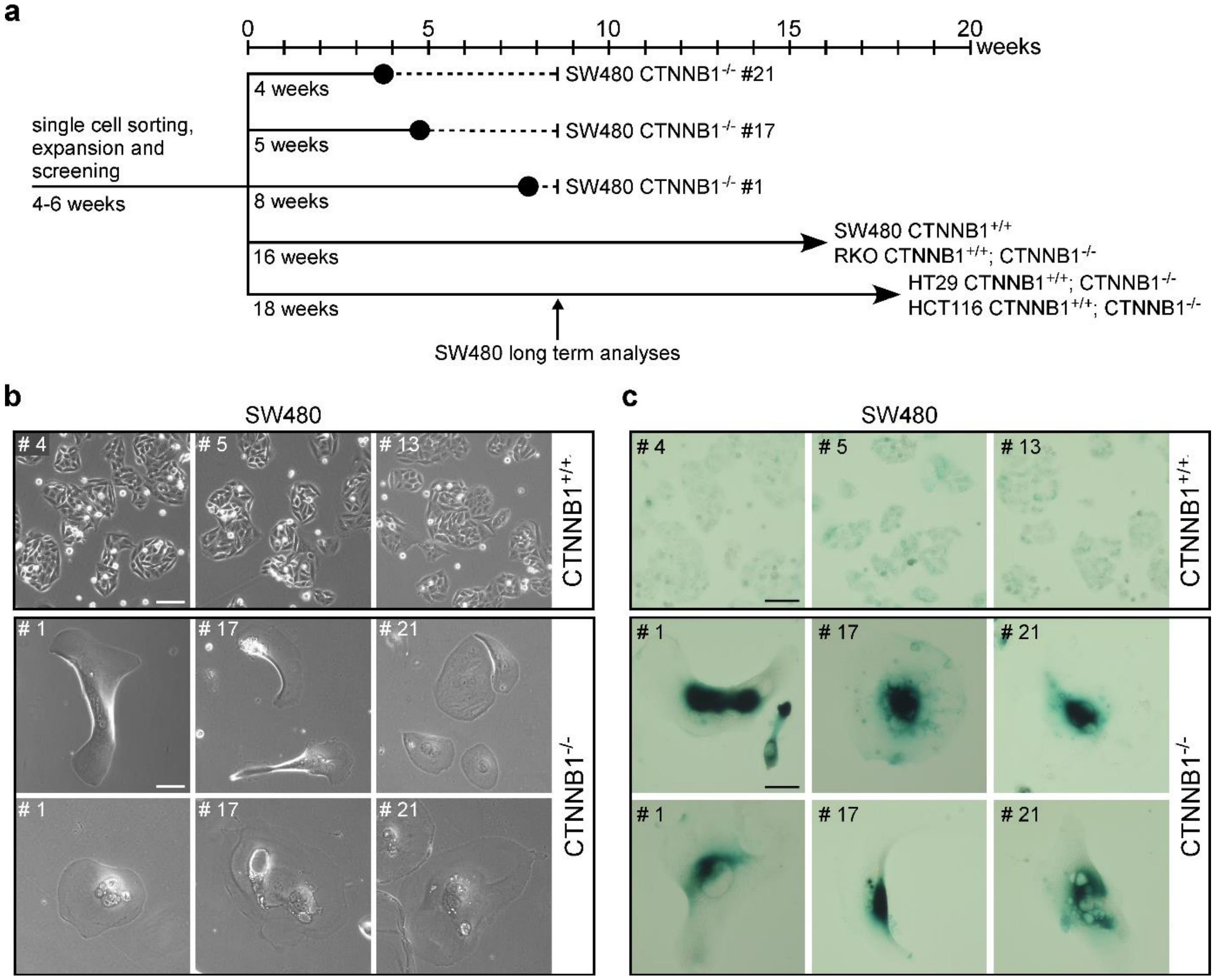

Suppl. Fig. S3: β-CATENIN-deficient SW480 cells enter senescence after long term cell culture. a Schematic representation of the cultivation times and growth behavior of *CTNNB1*$^{+/+}$ and *CTNNB1*$^{-/-}$ CRC cells as indicated. Large black dots represent the time points at which SW480 *CTNNB1*$^{-/-}$ cell clones stopped proliferating. Dashed lines show the additional time for which cell were kept in culture until analysis. Arrows denote that cells continue to proliferate. The vertical arrow indicates the time point at which all β-CATENIN-deficient SW480 cells were harvested and analyzed. **b** Representative micrographs showing *CTNNB1*$^{+/+}$ and *CTNNB1*$^{-/-}$ SW480 cell clones. Images from one of three independent biological replicates were taken approximately 16 weeks after expression of Cas9 and sgRNAs, and single cell-sorting. The scale bars represent 100 µm. **c** β-GALACTOSIDASE stainings of *CTNNB1*$^{+/+}$ and *CTNNB1*$^{-/-}$ SW480 cell clones performed 14 - 16 weeks after expression of Cas9 and sgRNAs, and single cell-sorting. The pictures are representative micrographs from one of three independent biological replicates. The scale bars represent 100 µm.

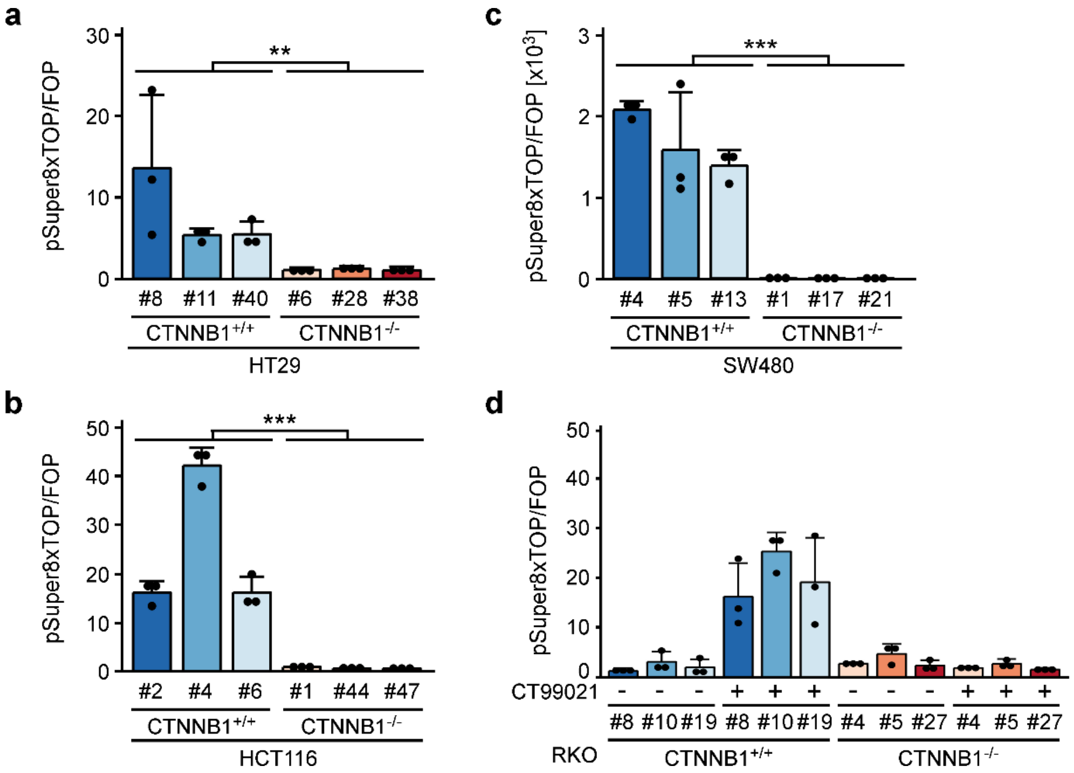

Suppl. Fig. S4: Transcriptional activity of the Wnt/β-CATENIN pathway is abolished in β-CATENIN-deficient HT29, HCT116, SW480, and RKO cells. a-d *CTNNB1*$^{+/+}$ and *CTNNB1*$^{-/-}$ HT29 (**a**), HCT116 (**b**), SW480 (**c**), and RKO cells (**d**) were cotransfected with expression vectors for *R. reniformis* and firefly luciferase reporter genes with wild type (pSuper8xTOPFlash) or mutant (pSuper8xFOPFlash) TCF/LEF binding sites in their promoter elements. RKO cells were additionally treated with CT99021 to stimulate Wnt pathway activity. Luciferase activities were determined 48 h post transfection. *R. reniformis* luciferase activities were used for normalization of firefly luciferase activities. The bar graphs show the ratios of normalized firefly luciferase activities from cells transfected with pSuper8xTOPFlash and with pSuper8xFOPFlash. Each dot represents an individual measurement. Error bars indicate SD (n=3). For statistical analysis LMM was performed.

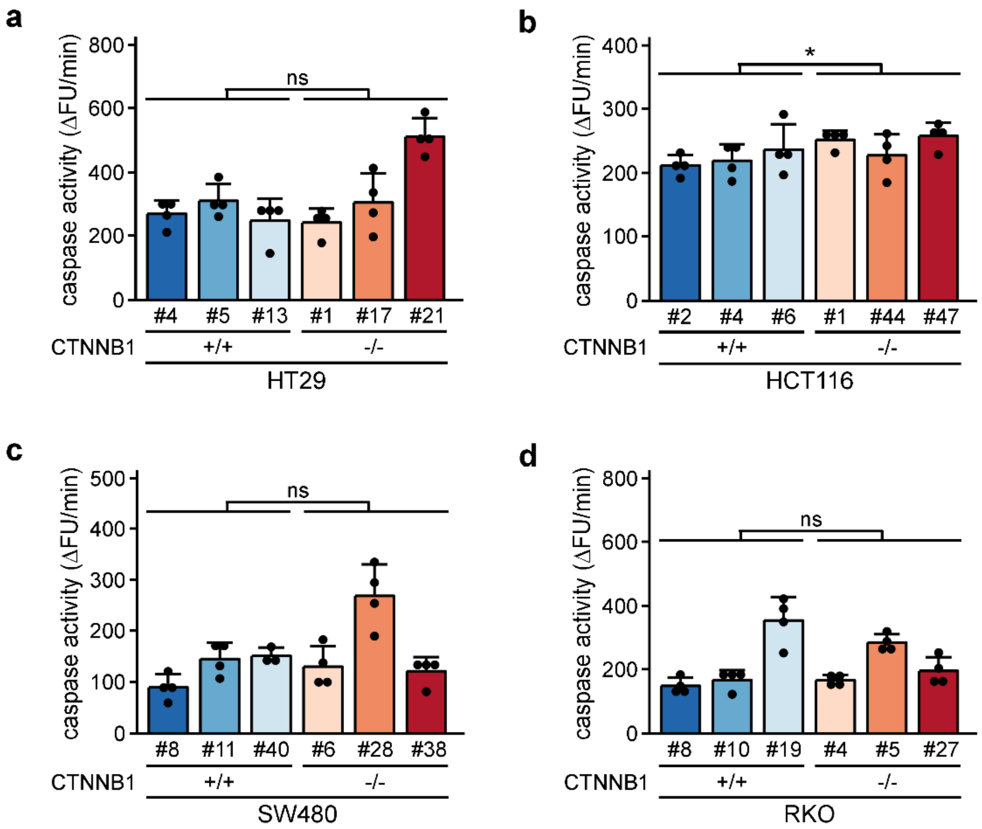

Suppl. Fig. S5: β-CATENIN-deficiency does not lead to increased apoptosis in HT29, HCT116, SW480, and RKO cells. a-d *CTNNB1*$^{+/+}$ and *CTNNB1*$^{-/-}$ HT29 (**a**), HCT116 (**b**), SW480 (**c**), and RKO (**d**) cells were seeded and incubated for 24 h. Upon cell lysis and preparation of whole cell extracts, caspase 3 activity was determined by a fluorimetric assay and kinetic measurements. The resulting changes in fluorescence units (ΔFU) per min are displayed in the bar plots. Each dot represents an individual measurement. Error bars indicate SD (n=3). For statistical analysis LMM was performed. ns: not significant.

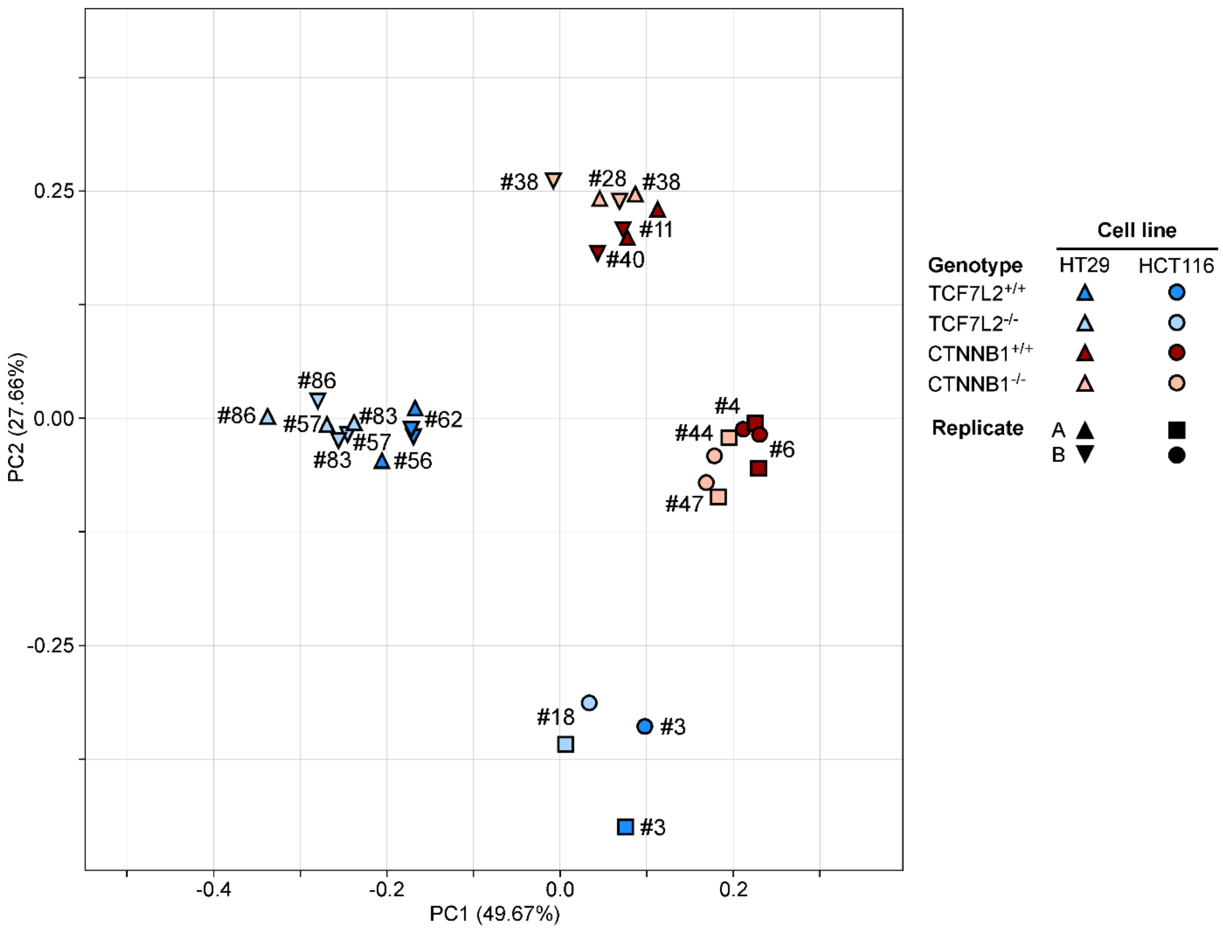

Suppl. Fig. S6: Principal component analysis (PCA) of transcriptome data derived from HT29 and HCT116 cell clones with biallelic WT (+/+) and mutant (-/-) *TCF7L2* and *CTNNB1* genes. Except for HCT116 TCF7L2 WT and mutant cells, two different cell clones were analyzed by RNA-seq for each cell line and genotype. Two independent biological replicates were performed (n=2).

Suppl. Fig. S7

Related to Figure 1b:

Gel 1

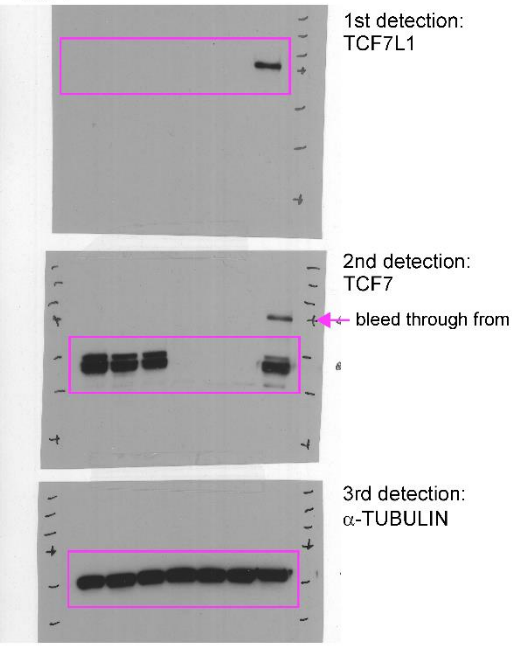

1st detection:
TCF7L1

2nd detection:
TCF7
← bleed through from 1st detection

3rd detection:
α-TUBULIN

Gel 2

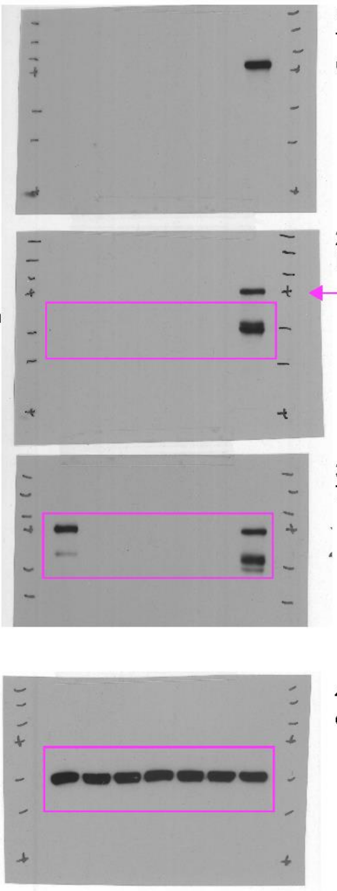

1st detection:
TCF7L1
(not used)

2nd detection:
LEF1
← bleed through from 1st detection

3rd detection:
TCF7L2

4th detection:
α-TUBULIN

Related to Figure 1c:

Gel 1

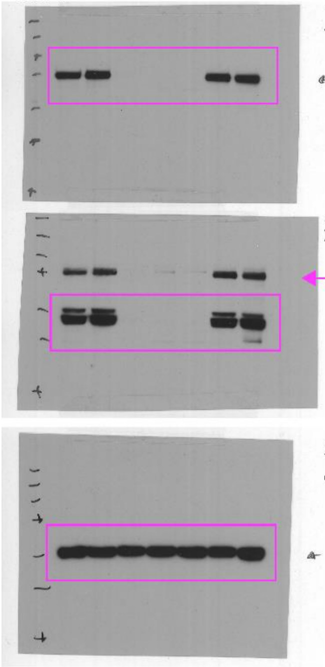

1st detection:
TCF7L1

2nd detection:
TCF7
← bleed through from 1st detection

3rd detection:
α-TUBULIN

Gel 2

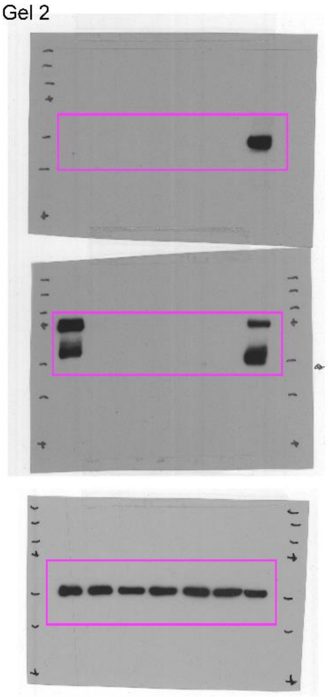

1st detection:
LEF1

2nd detection:
TCF7L2

3rd detection:
α-TUBULIN

Suppl. Fig. S7 continued →

Suppl. Fig. S7 continued:

Related to Figure 3b:

HT29

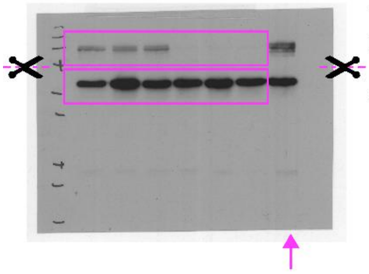

1st detection*: β-CATENIN & α-TUBULIN

extra lane with HT29 WT lysate

SW480

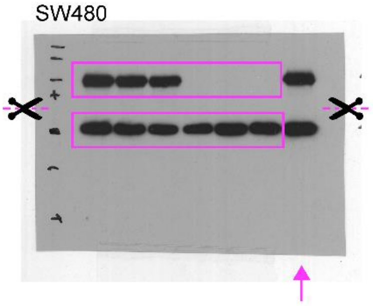

1st detection*: β-CATENIN & α-TUBULIN

extra lane with SW480 WT lysate

HCT116

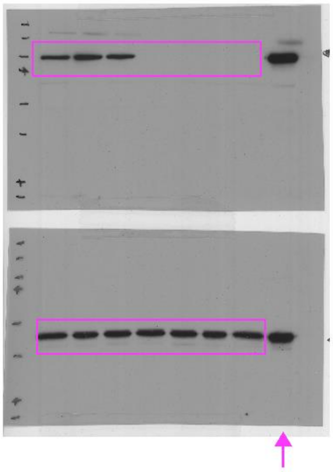

1st detection: β-CATENIN

2nd detection: GSK3β

extra lane with HCT116 WT lysate

RKO

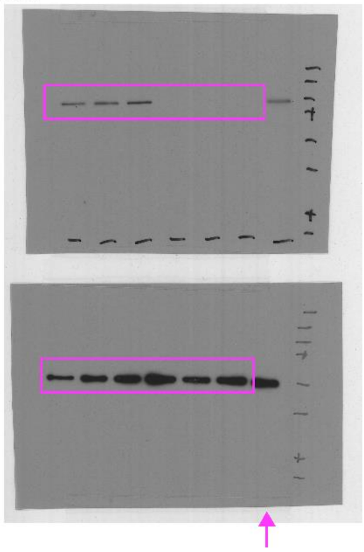

1st detection: β-CATENIN

2nd detection: α-TUBULIN

extra lane with RKO WT lysate

*: after the gel run and transfer to nitrocellulose, the membrane was cut horizontally below the 75 kDa marker (4th from top, marked with a cross). The top and bottom parts of the membrane were then processed separately for detection of β-CATENIN and α-TUBULIN. However, for signal detection, the two pieces of membrane were reassembled.

Suppl. Fig. S7: Full size images of Western blots shown in Figures 1 and 3 of the main manuscript. Pink frames highlight areas used for display in the final versions of the figures. When membranes were sequentially probed, the order of detection is given and bleed-through signals reappearing during subsequent rounds of the detection, are marked.

Supplementary Table S1: Cell lines used in this study

Cell line[*,#]	Source	Culture conditions
HCT116	Max-Planck-Institute for Immunology and Epigenetics (Freiburg, Germany)	DMEM with 4.5 g/l glucose, stabilized glutamine, sodium pyruvate, and 3.7 g/l $NaHCO_3$, supplemented with:<br>10 % (v/v) fetal calf serum<br>10 mM HEPES<br>1 % (v/v) MEM non-essential amino acids solution<br>1 % (v/v) penicillin/streptomycin<br>grown at 37 °C and 5 % CO_2.
HCT116 $TCF7L2^{+/+}$ clone 3[§]	derived from HCT116	
HCT116 $TCF7L2^{-/-}$ clone 33[§]	derived from HCT116	
HT29	German Cancer Research Center Cell Line Service (Heidelberg, Germany)	
HT29 $TCF7L2^{+/+}$ clone 56[§]	derived from HT29	
HT29 $TCF7L2^{-/-}$ clone 83[§]	derived from HT29	
LoVo	CLS Cell Lines Service GmbH (Eppelheim, Germany)	
LS174T	CLS Cell Lines Service GmbH (Eppelheim, Germany)	
LS411	Institute of Molecular Medicine and Cell Research (Freiburg, Germany)	
RKO	Institute of Molecular Medicine and Cell Research (Freiburg, Germany)	
SW403	ATCC, Manassas, Virginia, USA	
SW480	Max-Planck-Institute for Immunology and Epigenetics (Freiburg, Germany)	

* Cell line identity was determined by SNP-profiling at Multiplexion Inc. (Friedrichshafen, Germany).

[#] Cell lines were routinely tested for mycoplasma contamination using the MycoSensor PCR Assay Kit from Agilent (Agilent Technologies Deutschland GmbH, Waldbronn, Germany).

[§] generation of these cell lines was described before (Wenzel et al., 2020).

Supplementary Table S2: Summary of genome editing strategies and genotypes of cell clones generated in this study

Derived from HT29 *TCF7L2*$^{-/-}$ clone #83.7* (Wenzel et al., 2020)			
**targeted gene: *TCF7***			
**clone ID**	**sgRNAs used**	**genotype**	**allele 1/2**
#5	sgTCF7-1<br>sgTCF7-2	KO	∆ ENSE00003616545
#6	sgTCF7-1<br>sgTCF7-2	KO	∆ ENSE00003616545
#11	sgTCF7-1<br>sgTCF7-2	KO	∆ ENSE00003616545

* For inactivation of *TCF7*, HT29 *TCF7L2*$^{-/-}$ #83.7 cells were used. These cells are derived from HT29 *TCF7L2*$^{-/-}$ cell clone #83 by lentiviral transduction with a TCF7 cDNA construct which, however, is not expressed.

Derived from HCT116 *TCF7L2*$^{-/-}$ clone #33 (Wenzel et al., 2020)						
**clone ID**	**targeted gene: *TCF7***			**targeted gene: *TCF7L1***		
	**sgRNAs used**	**genotype**	**allele 1/2**	**sgRNAs used**	**genotype**	**allele 1/2**
#61	sgTCF7-1<br>sgTCF7-2	WT	WT	sgTCF7L1-1<br>sgTCF7L1-2	WT	WT
#58	sgTCF7-1<br>sgTCF7-2	KO	∆ ENSE00003616545	sgTCF7L1-1<br>sgTCF7L1-2	KO	209 bp deletion; nucleotides 28-128 of ENSE00000963602[a]
#59	sgTCF7-1<br>sgTCF7-2	KO	∆ ENSE00003616545	sgTCF7L1-1<br>sgTCF7L1-2	KO	272 bp deletion; nucleotides 1-78 of ENSE00000963602[a]
#60	sgTCF7-1<br>sgTCF7-2	KO	∆ ENSE00003616545	sgTCF7L1-1<br>sgTCF7L1-2	KO	209 bp deletion; nucleotides 28-128 of ENSE00000963602[a]

derived from HCT116 cells			
**targeted gene: *CTNNB1***			
**clone ID**	**sgRNAs used**	**genotype**	**allele 1/2**
#2	sgCTNNB1-1<br>sgCTNNB1-2	WT	WT
#4	sgCTNNB1-1<br>sgCTNNB1-2	WT	WT
#6	sgCTNNB1-1<br>sgCTNNB1-2	WT	WT
#1	sgCTNNB1-1<br>sgCTNNB1-2	KO	258 bp deletion; nucleotides 17-145 of ENSE00001643204[b]
#44	sgCTNNB1-1<br>sgCTNNB1-2	KO	258 bp deletion; nucleotides 17-145 of ENSE00001643204[b]
#47	sgCTNNB1-1<br>sgCTNNB1-2	KO	258 bp deletion; nucleotides 17-145 of ENSE00001643204[b]

continued →

derived from HT29 cells			
targeted gene: *CTNNB1*			
**clone ID**	**sgRNAs used**	**genotype**	**allele 1/2**
#8	sgCTNNB1-1<br>sgCTNNB1-2	WT	WT
#11	sgCTNNB1-1<br>sgCTNNB1-2	WT	WT
#40	sgCTNNB1-1<br>sgCTNNB1-2	WT	WT
#6	sgCTNNB1-1<br>sgCTNNB1-2	KO	258 bp deletion; nucleotides 17-145 of ENSE00001643204[b]
#28	sgCTNNB1-1<br>sgCTNNB1-2	KO	258 bp deletion; nucleotides 17-145 of ENSE00001643204[b]
#38	sgCTNNB1-1<br>sgCTNNB1-2	KO	258 bp deletion of nucleotides 17-145 of ENSE00001643204[b]

derived from SW480 cells			
targeted gene: *CTNNB1*			
**clone ID**	**sgRNAs used**	**genotype**	**allele 1**
#4	sgCTNNB1-1<br>sgCTNNB1-2	WT	WT
#5	sgCTNNB1-1<br>sgCTNNB1-2	WT	WT
#13	sgCTNNB1-1<br>sgCTNNB1-2	WT	WT
#1	sgCTNNB1-1<br>sgCTNNB1-2	KO	258 bp deletion; nucleotides 17-145 of ENSE00001643204[b]
#17	sgCTNNB1-1<br>sgCTNNB1-2	KO	258 bp deletion; nucleotides 17-145 of ENSE00001643204[b]
#21	sgCTNNB1-1<br>sgCTNNB1-2	KO	258 bp deletion; nucleotides 17-145 of ENSE00001643204[b]

derived from RKO cells			
targeted gene: *CTNNB1*			
**clone ID**	**sgRNAs used**	**genotype**	**allele 1/2**
#8	sgCTNNB1-1<br>sgCTNNB1-2	WT	WT
#10	sgCTNNB1-1<br>sgCTNNB1-2	WT	WT
#19	sgCTNNB1-1<br>sgCTNNB1-2	WT	WT
#4	sgCTNNB1-1<br>sgCTNNB1-2	KO	258 bp deletion; nucleotides 17-145 of ENSE00001643204[b]
#5	sgCTNNB1-1<br>sgCTNNB1-2	KO	258 bp deletion; nucleotides 17-145 of ENSE00001643204[b]
#27	sgCTNNB1-1<br>sgCTNNB1-2	KO	258 bp deletion; nucleotides 17-145 of ENSE00001643204[b]

[a] coordinates refer to the 5´-end of ENSE00000963602
[b] coordinates refer to the 5´-end of ENSE00001643204

Supplementary Table S3: Results of genotyping PCRs for all knock-out attempts of this study

Gene(s) targeted	TCF7		TCF7 / TCF7L1	
Cell line	HT29 TCF7L2 KO #83.7		HCT116 TCF7L2 KO #33	
total number of clones screened	37		61	
wild type	2	5 %	7 / 32	11 % / 52 %
heterozygous	22	59 %	15 / 16	25 % / 26 %
homozygous mutant	10	27 %	20 / 9	33 % / 15 %
unclear	3	8 %	19 / 4	31 % / 7 %

Gene targeted	CTNNB1							
Cell line	HT29		HCT116		SW480		RKO	
total number of clones screened	59		86		49		128	
wild type	17	29 %	24	28 %	12	24 %	44	34 %
heterozygous	7	12 %	24	28 %	20	41 %	51	40 %
homozygous mutant	16	27 %	31	36 %	7	14 %	21	16 %
unclear	19	32 %	7	8 %	10	20 %	12	9 %

Gene targeted	CTNNB1							
Cell line	LS411*		LS174T		SW403*		LoVo*	
total number of clones screened	17		105		26		11	
wild type	16	94 %	98	93 %	20	77 %	9	82 %
heterozygous	0	0 %	1	1 %	0	0 %	1	9 %
homozygous mutant	0	0 %	0	0 %	0	0 %	0	0 %
unclear	1	6 %	6	6 %	6	23 %	1	9 %

*: in case of these cell lines, the number of clones screened equals the number of clones which could be recovered from a total of four 96-well plates after transfection and single-cell sorting. This could indicate limited clonogenicity of these cell lines.

Supplementary Table S4: Colorectal cancer driver pathway mutations[1] in the cell lines used in this study

Cell line	MSI status[2]	CIMP status	APC	FBXW7	CTNNB1	TCF7	TCF7L1	TCF7L2	LEF1	KRAS	BRAF	PIK3CA	TP53	SMAD4	TGFBR2
HCT116	U	+	+/+	+/+	+/-	+/+	+/+	+/+	+/+	+/-	+/+	+/-	+/+	+/+	-/-
HT29	S	+	-/-	+/+	+/+	+/+	+/+	+/+	+/+	+/+	+/-	+/-	-/-	-/-	+/+
LoVo	U	-	-/-	+/+	+/+	+/-	+/+	+/-	+/+	+/-	+/+	+/+	+/+	+/+	-/-
LS174T	U	-	+/+	+/+	-/-	+/-	+/-	+/-	+/+	+/-	+/-	+/-	+/+	+/+	-/-
LS411	U	+	-/-	+/+	+/+	+/+	+/+	+/+	+/+	+/+	+/-	+/+	-/-	+/+	-/-
RKO	U	+	+/+	+/+	+/-[3]	+/+	+/-	+/-	+/+	+/+	+/-	+/-	+/+	+/+	+/+
SW403	S	-	-/-	+/+	+/+	+/+	+/+	+/+	+/+	+/-	+/+	+/-	-/-	+/+[4]	-/-
SW480	S	-	-/-	+/+	+/+	+/+	+/+	+/+	+/+	+/-	+/+	+/+	-/-	+/+[4]	-/-

[1]: Information about microsatellite instability (MSI), CpG island methylator phenotpye (CIMP), and mutational states of the genes listed were taken from Mouradov et al., 2014.

[2]: U - unstable; S - stable

[3]: one allele of *CTNNB1* carries a silent mutation in RKO cells (Mouradov et al., 2014)

[4]: despite the wild type status of *SMAD4* reported by Mouradov and colleagues (Mouradov et al., 2014) no SMAD4 protein can be detected in SW480 and SW403 cells (Woodford-Richens et al., 2001; Frey et al., 2022)

Supplementary Table S16: List of sgRNA target sequences (without PAM)

Gene	sgRNA	Location	Sequence (5` - 3`)
CTNNB1	sgCTNNB1-1	exon 7	CTCATCATACTGGCTAGTGG
CTNNB1	sgCTNNB1-2	intron 7	GGTACTCTGAATGTAAATCT
TCF7	sgTCF7-1	intron 3	GTGAGTGTGGCGAGTCCTGA
TCF7	sgTCF7-2	intron 4	CCTGGGGCTGTGCAAACTAA
TCF7L1	sgTCF7L1-1	exon 3	TTAAAGAACGCGCTGTCCTG
TCF7L1	sgTCF7L1-1	intron 3	CTGCTTGGGATCGGCGCAGA

Supplementary Table S17: List of oligonucleotide primers used in this study

Primers for genotyping		
**Gene**	**Forward primer (5` - 3`)**	**Reverse primer (5` - 3`)**
CTNNB1	GGACAAGTTGGATAGGGCCC	GCACACGAAACCCCTGTGA
TCF7	GCAAAGTCTTGGGGGCTAGT	GGGTCACCCATGGGATTTAGG
TCF7L1	GCTCACCCGCTCTTGCCTTTGT	GAGGACAACGTCGCCAACCCAG
**Primers for qRT-PCR**		
**Gene**	**Forward primer (5` - 3`)**	**Reverse primer (5` - 3`)**
ASCL2	TGACCTGGGGCGTAATAAAG	ACACAGGCTTCTCCCTAGCA
AXIN2	TGCTTTCGTGGAAATGACAG	AGGTGTGTGGAGGAAAGGTG
CTNNB1	ACTGGCAGCAACAGTCTTAC	GTGGCAAGTTCTGCATCATC
GAPDH	ACCACAGTCCATGCCATCACT	GTCCACCACCCTGTTGCTGTA
LEF1	ACAGATCACCCCACCTCTTG	TGAGGCTTCACGTGCATTAG
MYC	AAGAGGACTTGTTGCGGAAA	CTCAGCCAAGGTTGTGAGGT
RNF43	CTGCTACCAGAAACCCCAGG	CTGCGGTGTCAGAACTCCAT
TCF7	AGCCAAGGTCATTGCAGAGT	GTGGTGGATTCTTGGTGCTT
TCF7L1	GGGTACCCCTTCCTGATGAT	GATGGTGACCTCGTGTCCTT
TCF7L2	AGAAAAGAAGAAGCCCCACA	CGGGCCAGCTCGTAGTATT
TERT	CTACGGCGACATGGAGAACA	AGAGATGACGCGCAGGAAAA

Supplementary Table S18: List of antibodies used in this study

Antigen	Species of origin	Dilution	Supplier (catalogue no.)
α-TUBULIN	mouse	1:10 000	Sigma-Aldrich (T9026)
β-CATENIN	rabbit	1:1000	Cell Signaling Technology (#9581)
LEF1	rabbit	1:1000	Cell Signaling Technology (#2230)
TCF7	rabbit	1:1000	Cell Signaling Technology (#2203)
TCF7L1	rabbit	1:1000	Cell Signaling Technology (#2883)
TCF7L2	rabbit	1:1000	Cell Signaling Technology (#2565)

References

Frey, P., Devisme, A., Rose, K., Schrempp, M., Freihen, V., Andrieux, G., Boerries, M., and Hecht, A. (2022). SMAD4 mutations do not preclude epithelial-mesenchymal transition in colorectal cancer. Oncogene *41*, 824-837.

Mouradov, D., Sloggett, C., Jorissen, R.N., Love, C.G., Li, S., Burgess, A.W., Arango, D., Strausberg, R.L., Buchanan, D., and Wormald, S., et al. (2014). Colorectal cancer cell lines are representative models of the main molecular subtypes of primary cancer. Cancer Res *74*, 3238-3247.

Wenzel, J., Rose, K., Haghighi, E.B., Lamprecht, C., Rauen, G., Freihen, V., Kesselring, R., Boerries, M., and Hecht, A. (2020). Loss of the nuclear Wnt pathway effector TCF7L2 promotes migration and invasion of human colorectal cancer cells. Oncogene *39*, 3893-3909.

Woodford-Richens, K.L., Rowan, A.J., Gorman, P., Halford, S., Bicknell, D.C., Wasan, H.S., Roylance, R.R., Bodmer, W.F., and Tomlinson, I.P. (2001). SMAD4 mutations in colorectal cancer probably occur before chromosomal instability, but after divergence of the microsatellite instability pathway. Proc Natl Acad Sci U S A *98*, 9719-9723.
